# Autologous and allogeneic hematopoietic cell transplantation in children and adults with high-risk anaplastic large cell lymphoma

**DOI:** 10.3389/fonc.2026.1785566

**Published:** 2026-04-22

**Authors:** Natalie L. Smith, Corinne Summers, Ted Gooley, Andrei Shustov, Rachel Salit, Rebecca A. Gardner, Ann Dahlberg, Marie Bleakley, Mohamed L. Sorror, Leona Holmberg, Paul A. Carpenter, Brenda M. Sandmaier, David Maloney, Monica S. Thakar

**Affiliations:** 1 Fred Hutchinson Cancer Center, Seattle, WA, United States; 2 Department of Pediatrics, University of Washington School of Medicine, Seattle, WA, United States; 3 Department of Medicine, University of Washington School of Medicine, Seattle, WA, United States; 4 Cancer and Blood Disorders Center, Seattle Children's Hospital, Seattle, WA, United States

**Keywords:** anaplastic large cell lymphoma, hematopoietic cell transplantation, autologous transplantation, allogeneic transplantation, lymphoma

## Abstract

**Introduction:**

Both autologous and allogeneic hematopoietic cell transplantation (HCT) have been used to treat high-risk anaplastic large cell lymphoma (ALCL) that has failed conventional chemotherapy. However, many patients are not able to fully eradicate disease prior to HCT, making the role of either form of HCT unclear. The aim of this study was to collect characteristics and outcomes of patients who underwent either autologous and/or allogeneic HCT and determine the role that disease status at HCT plays in overall survival (OS).

**Methods:**

To address this, we collected data on 41 patients with ALCL that underwent autologous (n=24) and/or allogeneic (n=17) HCT at our center between 1997-2020.

**Results:**

The 5-year estimated progression-free survival (PFS) and OS for the autologous HCT group was 40% (95% confidence interval, 20-59%) and 53% (31-71%), while for the allogeneic HCT group it was 65% (38-92%) and 65% (38-82%), respectively. For transplants performed in 2009 or later, 5-year OS was 91% (51-99%) in the allogeneic HCT group, and 51% (23-73%) in the autologous HCT group. Notable was that for those who entered transplant with detectable disease, 5-year OS was 50% (15-77%) with allogeneic HCT whereas it was 27% (7-54%) for autologous HCT.

**Discussion:**

These data confirm that HCT is a potentially curative therapy, and allogeneic HCT may have a survival advantage over autologous HCT when there is detectable disease prior to HCT.

## Introduction

Anaplastic large cell lymphoma (ALCL), a peripheral T-cell lymphoma with a characteristic phenotype of CD30 expression, is often sensitive to conventional chemotherapy yielding long-term overall survival (OS) as high as 80-90% (1). However, those with negative expression of anaplastic lymphoma kinase (ALK), age over 40, central nervous system (CNS) involvement, high International Prognostic Index score, and failure of disease response following brentuximab vedotin (BV, anti-CD30 monoclonal antibody-toxin therapy) have inferior OS (2–4). While regimens incorporating BV provide a survival advantage, there remains a subset of high-risk patients who require additional intensified therapy. Autologous hematopoietic cell transplantation (autoHCT) has been established as an effective consolidative treatment for high-risk ALCL, including those with ALK-negative disease (5–7). However, in the relapsed/refractory setting, autoHCT is associated with poor progression-free survival (PFS) (8–11). Allogeneic HCT (alloHCT) may provide additional benefit due to the immunological response attributed to the graft-versus-lymphoma (GVL) effect (10, 12, 13) but outcomes may be diminished by toxicity and graft-versus-host disease (GVHD) (14, 15). Reduced-intensity conditioning (RIC) regimens have been shown to retain GVL while decreasing non-relapse mortality (NRM) and in combination with targeted agents like BV, can lead to excellent OS (16, 17). However, it is unclear if either approach is effective at treating patients who present to HCT with persistent disease. Here we provide results from what we believe is the largest single-center cohort study of patients treated with HCT for high-risk ALCL.

## Materials and methods

### Study design

Patients underwent HCT at the Fred Hutchinson Cancer Center (FHCC) between 1997–2020 for treatment of ALCL and were considered high risk due to stage at diagnosis, ALK status, treatment sensitivity, and/or relapse status. All diagnoses were confirmed by FHCC pathology. This study was approved by the local FHCC Institutional Review Board and data were retrospectively collected through FHCC’s HCT database and/or patients’ medical records. Each patient had previously signed an institutional consent form authorizing use of their medical records.

### Frontline treatment, HCT protocols and disease characteristics

Frontline treatment prior to HCT and choice of auto vs allo HCT, donor for alloHCT, and conditioning regimen were made based on the discretion of the treating physician. Conditioning-regimen intensity was defined as myeloablative or RIC as previously described (18, 19). Non-myeloablative regimens were included in the RIC category. Decision-making regarding HCT regimens, donors, and timing of these treatment modalities was not available as part of this retrospective data collection. HCT co-morbidity index (HCT-CI) (20) was determined for the allo cohort; the auto cohort was limited by data availability and therefore not reported. Additional data points collected included ALK expression status, CNS status, and prior lines of therapy.

### Disease status definitions

Disease status was categorized as chemosensitive (partial response or complete response to prior therapy), primary induction failure, or refractory to any prior therapy. Complete remission (CR) was defined as full response to therapy, defined as no evidence of disease by clinical or imaging modalities, which included PET CT when available, but did not exclude the presence of microspcopic/low-level disease not appreciated by imaging. First complete remission (CR1) denotes the first achievement of CR; with CR2 and so-forth denoting CR following a relapse event. Partial response (PR) was defined as residual evidence of disease by clinical or imaging modalities. Disease progression was defined as increased disease level following treatment. Refractory disease was defined as lack of disease level reduction or increase following treatment.

### HCT cohorts

Patients were subdivided by type of HCT received: either single autoHCT (auto cohort), or single alloHCT (+/- preceded by autoHCT; allo cohort). For purpose of analysis, patients in this last category were assessed for outcomes following alloHCT, and any prior autologous HCT was counted as a “prior line of therapy.” Patients in this last category included those having either a planned/upfront auto-then-alloHCT strategy or those who relapsed after autoHCT and then later underwent alloHCT.

### HCT era definition

Subgroup analyses included era of HCT (pre-2009 vs 2009 or later), ALK status, and CR v. no CR. The two eras of HCT (1997–2008 and 2009-2020) were chosen to be consistent with the timepoint when WHO guidelines shifted to using ALK expression to classify mature T cell lymphomas (21).

### Primary and secondary outcomes and statistical considerations

The primary objective was to estimate the probability of OS as a function of time in each cohort. Additional objectives included estimating the probabilities of PFS (alive without progression/relapse), NRM (death without prior relapse/progression), and relapse/progression as a function of time in each cohort. The Kaplan-Meier method was used to obtain point estimates for OS and PFS, while cumulative incidence (CI) estimates were used to summarize NRM and relapse/progression. Relapse/progression was considered a competing risk for NRM and NRM was considered a competing risk for relapse/progression. Additional secondary outcomes included acute and chronic GVHD. Since all patients had complete follow-up beyond 100 days, acute GVHD was summarized using simple proportions. Cox regression was used to summarize the difference between autoHCT and alloHCT groups after categorizing transplants into CR or no CR. Chronic GVHD was scored based on NIH criteria and the probability of chronic GVHD was obtained from a cumulative incidence estimate, with relapsed before chronic GVHD and death considered competing risks (22).

## Results

### Patient characteristics

Forty-one patients met criteria for inclusion (**Table 1**). Over half of HCTs were performed in 2009 or later (auto, 62.5%; allo, 65%). The alloHCT cohort contained six patients with prior autoHCTs, of which four were planned tandem auto-allo HCT. In this group of six, median time between auto and allo HCT was 2.7 months (range, 1.7–66.2). The autoHCT cohort included 58% of patients with stage III or IV disease at diagnosis, and 47% were in the alloHCT cohort. ALK expression status was known for most patients and was predominantly ALK-negative in the autoHCT group (75%) compared to 41% in the alloHCT group. The median HCT-CI score for the alloHCT cohort was 3 (range, 0-6) (**Supplementary Table 1**).

**Table 1:** Patient and disease characteristics.

HCT cohort	AutoHCT (n=24)	AlloHCT (n=17)
Characteristic	n (%)	n (%)
Female Sex	10 (42)	8 (47)
Year of HCT		
Before 2009	9 (37.5)	6 (35)
2009 or later	15 (62.5)	11 (65)
Ann Arbor Stage III or IV	14 (58)	8 (47)
ALK expression status		
Positive	5 (21)	8 (47)
Negative	18 (75)	7 (41)
Missing	1 (4)	2 (12)
CD3 expression status		
Positive	11 (46)	4 (24)
Negative	12 (50)	11 (65)
Missing	1 (4)	2 (12)
CNS Involvement	0	3 (18)
< 12 months from diagnosis to HCT	10 (42)	6 (35)^a^
Disease status at HCT		
CR1	5 (20)	2 (12)
CR2	8 (33)	5 (29)
CR3	0	2 (12)
Partial Response	7 (29)	5 (29)
Progressive/Refractory	4 (17)	3 (18)
Brentuximab vedotin pre-HCT	8 (33)	6 (35)
ALK-inhibitor pre-HCT	2 (8)	2 (12)
Prior Auto HCT	–	6 (35)
Characteristic	Median (Range)	Median (Range)
Age at Transplant	51 (22-67)	30 (3-62)
<18 years of age	0 (0)	4 (24)
Prior Lines of Therapy	2 (1-6)	4 (2-7)
Months from diagnosis to HCT	14 (5-182)	13 (7-76)

^a^ In the alloHCT cohort, <12 months from diagnosis to HCT refers to the time interval between diagnosis and alloHCT. This does not refer to timing in reference to any preceding autoHCT.

Auto, autologous HCT; allo, allogeneic HCT; HCT, hematopoietic cell transplantation

The median number of prior lines of therapy for the autoHCT cohort was 2 (range, 1-6) whereas for the alloHCT cohort it was 4 (range 2-7). Proportions of patients who received BV or an ALK inhibitor are listed in **Table 1**; for both cohorts, those who received ALK-inhibitor also received BV. For the patients who received BV-containing regimens, 88% (7/8) achieved CR or PR prior to autoHCT. There was a median of 14.2 months (range, 5.2-181.6) from diagnosis to HCT for the autoHCT cohort, with 42% of patients undergoing autoHCT within 1 year of diagnosis. The median time to HCT in the alloHCT cohort was 12.8 months (range, 6.5-76.2) with 35% of patients receiving an alloHCT within 1 year of diagnosis. In both cohorts, most patients were transplanted in CR. The proportion of patients with a PR or progressive/refractory disease at time of HCT was similar between auto vs alloHCT cohorts. Additionally, the proportion of patients entering alloHCT with progressive/refractory disease declined from 50% before 2009 to 0% after 2009 (**Supplementary Table 1**).

### HCT characteristics

In the auto cohort, there was a change in conditioning regimens based on HCT era, with the regimen consisting of cyclophosphamide/12 Gy total body irradiation (TBI) becoming less common after 2009 (**Supplementary Table 2**). Most patients undergoing alloHCT used RIC conditioning (59%), with the most frequent regimen consisting of cyclophosphamide/fludarabine/200 cGy TBI (**Supplementary Table 1**). Among allogeneic donor sources, 47% received human leukocyte antigen (HLA)-matched unrelated donors and 29% HLA-matched sibling donors (**Table 2**). The predominant stem cell source in alloHCT was peripheral blood stem cells (76%).

**Table 2:** HCT characteristics and outcomes.

HCT Cohort	AutoHCT (n=24)	AlloHCT (n=17)
Characteristic	n (%)	n (%)
HCT donor type		
Unrelated	–	8 (47)
Matched related	–	5 (29)
Haploidentical related	–	3 (18)
Unrelated cord blood unit	–	1 (6)
HLA matching		
10/10	–	12 (71)
9/10	–	1 (6)
Haploidentical	–	3 (17)
Mismatched cord blood unit	–	1 (6)
HCT cell source	–	
Peripheral blood	–	13 (76)
Bone marrow	–	3 (18)
Cord blood unit	–	1 (6)
Relapse/Progression	13 (54)	5 (42)
Achieved CR after HCT^a^	6 (55)	4 (50)
Maintained CR after HCT^b^	5 (21)	8 (47)
Maintenance therapy Post-HCT	8	0
Brentuximab	3	–
Other	5	–
Acute GVHD		
Grade II	–	14 (82)
Grade III or IV	–	0
Chronic GVHD (n=11)	–	7 (64)
Peak grade		
Unknown	–	1 (14)
Mild	–	1 (14)
Moderate	–	2 (29)
Severe	–	3 (43)
Characteristic	Median (Range)	Median (Range)
Years of Survivor Follow-Up	13.3 (1.4-24.3)	14.6 (5.9-16.2)
Months to Relapse/Progression^c^	4.2 (0.9-40.3)	2.5 (1.4-3.9)

^a^ If not CR at time of transplant. Population at risk for auto cohort, n=11; allo cohort, n=8.

^b^ If in CR at time of transplant.

^c^ Among those who relapsed/progressed.

autoHCT, autologous HCT; alloHCT, allogeneic HCT; CR, complete remission; GVHD, graft-vs-host disease.

### Autologous cohort HCT outcomes

**Figure 1:**
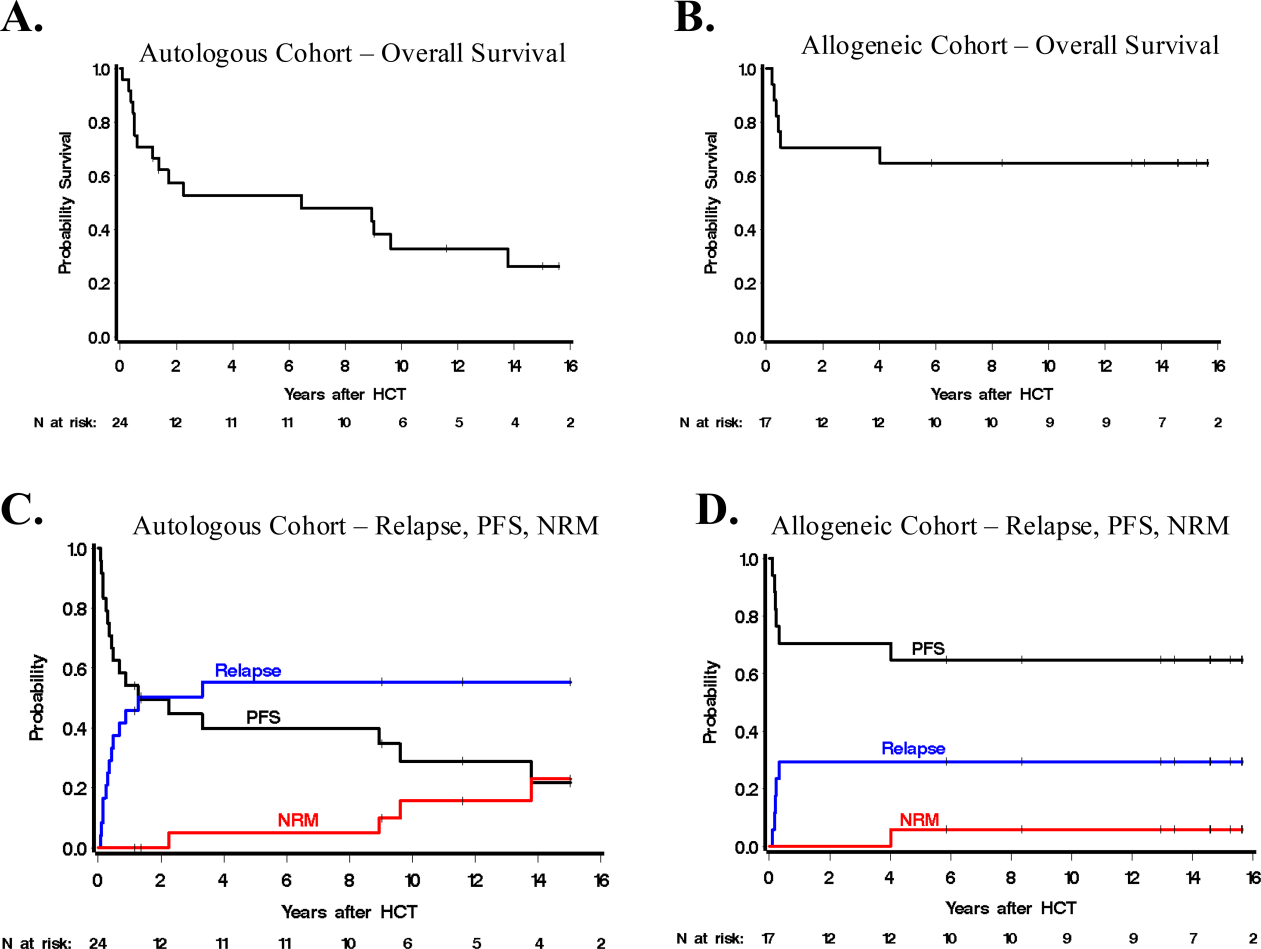
Overall survival estimates for **(A)** autologous HCT cohort and **(B)** allogeneic HCT cohort. Estimates for Progression Free Survival (PFS), Relapse or Progression, or Non-Relapse Mortality (NRM) for **(C)** autologous HCT cohort and **(D)** allogeneic HCT cohort. Estimates at 5-years denoted with dashed line.

**Figure 2:**
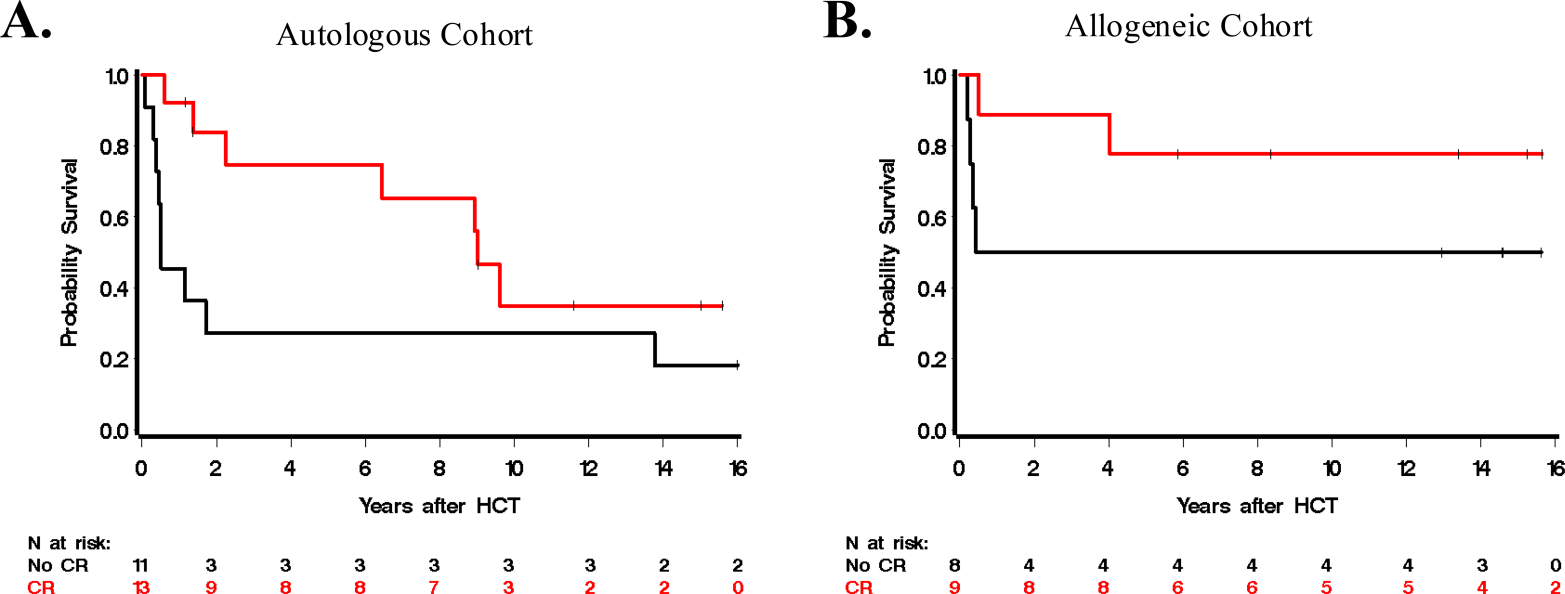
Overall survival estimates based on complete remission (CR) status at time of HCT for **(A)** autologous HCT cohort and **(B)** allogeneic HCT cohort.

**Figure 3:**
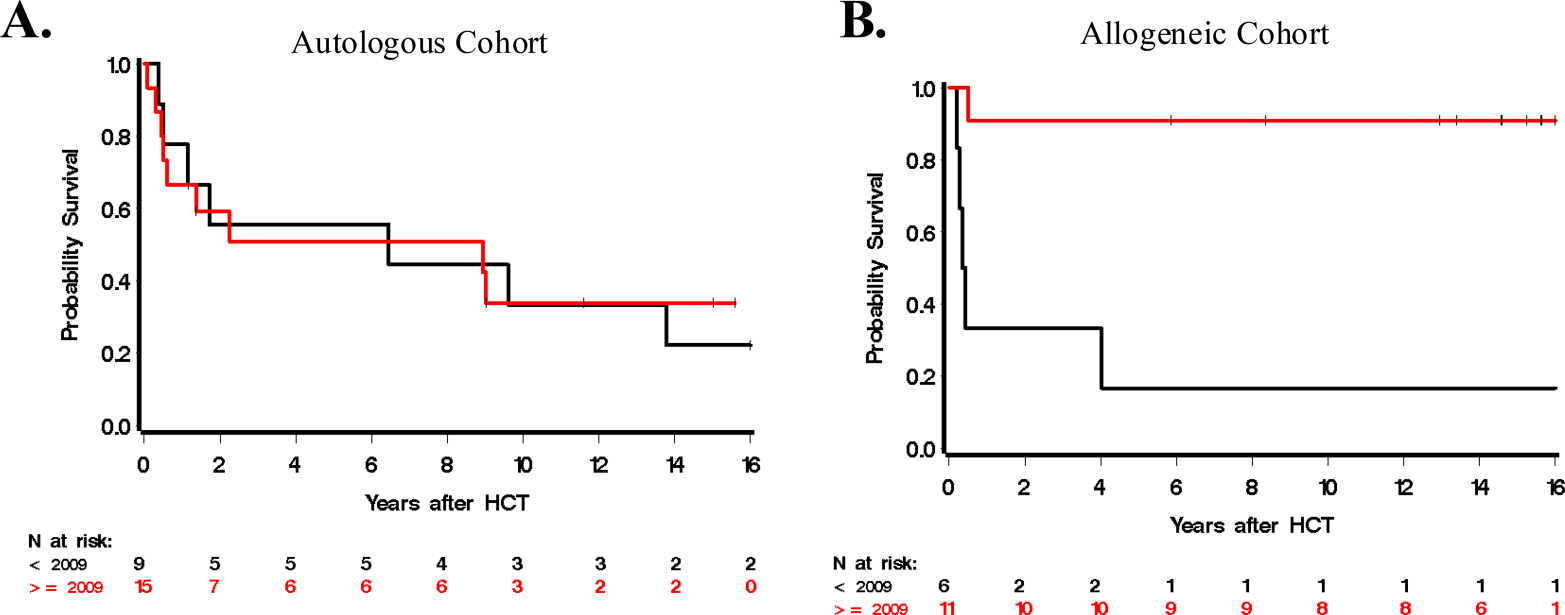
Overall survival estimates based on year of HCT for **(A)** autologous HCT cohort and **(B)** allogeneic HCT cohort.

Twenty-four patients were treated with autoHCT only. With a median follow-up time of 13.3 years among 8 survivors (range, 1.2-24.3), the 5-year OS was 53% (95% confidence interval (CI): 31-71%; **Figure 1**). Thirteen patients experienced relapse/progression following HCT, leading to 1- and 5-year point estimates of 46% (95% CI: 25-64%) and 55% (95% CI: 32-73%), respectively. PFS at 1 and 5 years were 54% (95% CI: 33-71%) and 40% (95% CI: 20-59%), respectively. Among 11 patients not in CR at time of HCT, six (55%) achieved CR after HCT, though three of these six patients eventually relapsed at 127, 320, and 461 days post-HCT. In total, eight of these 11 patients not in CR at time of transplant relapsed/progressed at a median of 127 days (range 27–461 days) after autoHCT. There were 4 NRM events in the autoHCT group. For the entire cohort, 1- and 5-year OS was 71% (95% CI: 57-85%) and 53% (95% CI: 31-71%), respectively. For those transplanted in CR, 1- and 5-year estimates of OS were 92% (95% CI: 57-99%) and 75% (95% CI: 40-91%), respectively, and for patients not in CR at HCT, 1- and 5-year OS estimates were 45% (17-71%) and 27% (7-54%), respectively (**Figure 2**). Estimates of OS as a function of HCT era (pre-2009 and 2009 and later) are summarized in **Figure 3**. In the autoHCT cohort, five of six patients with positive ALK-expression status died, while 10 of 17 who were negative died. The 1- and 5-year point estimates for OS in the ALK-negative group were 76% (95% CI: 49-90%) and 56% (95% CI: 29-77%), respectively.

### Allogeneic cohort HCT outcomes

A total of 17 patients were treated with alloHCT (with six having received prior auto). The median follow-up time amongst 10 survivors was 14.6 years (range 5.9-16.2), and the 5-year point estimate of OS was 65% (95% CI: 38-82%; **Figure 1**). There were five relapse/progression events among the 17 patients, and there were two deaths without relapse/progression at 4.0 and 16.2 years due to bronchiolitis obliterans syndrome and metastatic sarcoma, respectively. The median time of progression or relapse was not reached; among the 5 who progressed or relapsed, all did so within 117 days of alloHCT, and as a result, estimates of relapse/progression were 29% (95% CI: 10-52%) at all time points beyond 117 days post-alloHCT. Estimates of PFS at 1 and 5 years were 71% (95% CI: 43-87%) and 65% (95% CI: 38-82%), respectively. Of the eight patients not in CR at time of HCT, four (50%) went on to achieve CR after HCT. All patients (n=5) who received BV-containing regimens achieved a CR or PR prior to HCT.

Among the nine patients who received alloHCT in CR, there were 3 deaths (at 0.5, 4.0, and 16.2 years after HCT) and 1- and 5-year OS was 89% (95% CI: 43-98%) and 78% (95% CI: 36-94%), respectively. Among the eight transplanted not in CR, four have died, all before one year from HCT (1-year point estimate 50%; 95% CI: 15-77%; **Figure 2**) and each with a history of prior relapse. Five-year estimates of OS after or before 2009 were 91% (95% CI: 51-99%) and 17% (95% CI: 1-52%; **Figure 3**), respectively. Among seven patients who were ALK-negative, only one died, at 0.5 years. Three of eight ALK-positive patients died, at 0.2, 0.3, and 0.3 years.

### Outcomes based on CR status

Twenty-two of 41 patients were transplanted in CR. The 1- and 5-year estimates of OS were 91% (95% CI: 68-98%) and 76% (95% CI: 51-89%), respectively, for those transplanted in CR and 47% (95% CI: 24-67%) and 37% (95% CI: 17-57%), respectively, for those transplanted not in CR. For those transplanted in CR, the hazard ratio (HR, alloHCT vs. autoHCT) of mortality was 0.35 (95% CI: 0.07-1.69) and for those transplanted not in CR, HR 0.44 (0.20-2.08).

### GVHD in the AlloHCT cohort

GVHD prophylaxis regimens for alloHCT recipients are listed in **Table 2**. Grade II acute GVHD was seen in 82% of patients, but none had grades III or IV. There were 7 cases of chronic GVHD, with 5 patients relapsing and/or dying without chronic GVHD, and the estimated probability of chronic GVHD at one year was 41% (17-64%).(**Supplementary Table 1**).

## Discussion

This retrospective analysis of 41 patients with ALCL represents the largest single-center, transplant-focused study published to date. With a median follow-up among 18 transplant survivors of roughly fourteen years, we report excellent survival rates with low NRM. These data add to the body of literature confirming that HCT is an efficacious treatment strategy conferring encouraging survival for patients with high-risk ALCL (reviewed in **Supplementary Table 3**).

The observational nature of this study limits the ability to compare the autoHCT and alloHCT cohorts directly, but we observed interesting characteristics that differ between the two groups. The autoHCT cohort consisted of older patients who were more likely to be Ann Arbor stage III or IV and have ALK-negative disease. There were no pediatric patients in the autoHCT cohort, demonstrating that pediatric patients with relapsed disease were preferentially referred to alloHCT. Patients undergoing autoHCT were more likely to go to HCT sooner, with lower numbers of prior lines of therapy than the alloHCT group, though had similar numbers receiving BV and/or an ALK inhibitor. Both groups had changes in HCT conditioning at our center after 2009, with autoHCT transitioning away from TBI-based regimens and alloHCT increasing the use of RIC regimens. This timing also coincides with the introduction of BV (23). Estimated 5-year OS was 53% and 65% for autoHCT and alloHCT, respectively, although autoHCT had a higher relapse/progression rate (55% versus 29% at 5 years). Of the patients who entered HCT not in CR, 55% achieved CR after autoHCT, compared to 50% after alloHCT.

Of the 13 patients who experienced relapse after autoHCT, one was ultimately salvageable and survived until last follow-up at 15.6 years **Supplementary Table 2**). Furthermore, CR could be achieved in six of the 11 patients who entered autoHCT with partially responsive or progressive disease; however, this CR was durable for only for three patients (24.3, 18.8 and 18.1 years, respectively) and the other three patients ultimately relapsed at a median of 320 days after autoHCT. This suggests the importance of good disease control pre-HCT. Previous work evaluating long-term survival for adults with ALCL treated with various approaches including autoHCT found that ALK-negative status and age over 40 were poor prognostic factors (2). As our autoHCT cohort had a median age of 51 years and a majority were ALK negative, it is possible that this subgroup was biased against favorable outcomes.

Long-term OS was attainable for patients that underwent alloHCT (**Figure 1**). The 5-year PFS was 50% in this cohort, which supports the notion that once remission has been achieved after alloHCT in ALCL, it is typically long-standing. This finding concurs with data reported by the ALCL-Relapse trial, which investigated the use of alloHCT in children with first-relapse ALCL and found favorable OS 83% and EFS 81% at 5 years (24). In our study, the 5-year OS for allogeneic transplants that occurred after 2009 was 91% compared to 17% for transplants that occurred prior to 2009. This apparent improvement in OS is directly related to a decrease in the frequency of relapses (4 of 6 prior to 2009, 1 of 11 after 2009), suggesting the important role of ALK-inhibitors and BV in establishing pre-HCT disease control in this later cohort. Failure to achieve CR after treatment with BV in a relapsed/refractory ALCL cohort has been found to be a poor prognostic factor, though HCT following partial response to BV was associated with improved OS (25). The six patients in our study who received BV achieved PR/CR, supporting this concept. One patient who received BV due to disease progression after auto-HCT went on to achieve CR, was further treated with alloHCT, and remained in CR at last follow-up 7.3 years after alloHCT. Comparatively, all twelve patients who did not receive BV pre-HCT ultimately relapsed.

A notable outcome of this alloHCT cohort was the lack of relapse or progression events after the first 200 days following HCT. Of the five acute relapses that occurred, four patients were in a non-CR status at time of alloHCT. One potential contributor to long-term disease control for these alloHCT recipients was the GVL effect, as GVHD and GVL are immunologically related. Others have also noted the benefit of immune modulation for ALCL. Specifically, donor lymphocyte infusions led to disease response following post-HCT relapse with some evidence of improved OS in T cell lymphomas (26–28).

Additional factors that can enhance survival rates after HCT include improved supportive care practices and less toxic conditioning regimens. However, similar to our study, it has been reported that when using alloHCT to treat ALCL, rates of NRM are typically low and not demonstrably different between myeloablative and RIC regimens (29). The NRM of our entire alloHCT cohort was very low, with one patient transplanted before 2009 who died at four years after HCT from bronchiolitis obliterans syndrome. This suggests that the improved survival seen after 2009 can be best explained by improvements in pre-HCT therapies to improve disease control and therefore promote lower disease burden at time of HCT.

Important limitations of this study include the small sample size and heterogeneity of pre-HCT clinical factors. Due to the retrospective design of this study, wherein HCT strategy was determined by the treating physician based on the plurality of individual factors, intergroup comparison of autoHCT versus alloHCT outcomes is not appropriate. The post-HCT treatment course is not captured here and thus potential influences on outcomes from prophylactic and pre-emptive therapies after HCT cannot be considered. Furthermore, this was a retrospective analysis and prospective studies could provide additional insight into direct comparisons between autoHCT vs alloHCT for high-risk patients. Additionally, further study of autoHCT-then-alloHCT patients, particularly outcomes for those with planned tandem HCTs separated from those with failed autoHCT who went on to alloHCT, would be informative, as these two distinct strategies are grouped together and studied within the larger alloHCT cohort. Lastly, there is intrinsic bias introduced in cohort studies of HCT recipients wherein patients who could not receive HCT for a variety of reasons including severity of disease, co-morbidities, or lack of allograft availability were not considered by this analysis.

We believe that our study is the largest reported to date from a single center and supports the benefit of both autologous and allogeneic HCT for patients with ALCL especially in the BV era. Allogeneic HCT offers the benefit of GVL, which is suggested by the low incidence of relapse and a number of patients who experienced chronic GVHD. Prospective studies are necessary to further understand which patients would benefit from which HCT type.

## Data Availability

The raw data supporting the conclusions of this article will be made available by the authors, without undue reservation.
