# Supplementary material for: Autologous and allogeneic hematopoietic cell transplantation in children and adults with high-risk anaplastic large cell lymphoma
Source: Front Oncol. 2026 Apr 22;16:1785566. doi: 10.3389/fonc.2026.1785566 (PMC13147282; doi:10.3389/fonc.2026.1785566)
Supplement: Supplementary file 1 [file Table1.docx]

**Supplementary Table 1 (S1).** Detailed Characteristics of Allogeneic Cohort

| ID | Year of HCT | Age at AlloHCT  (Years) | Ann Arbor Stage at Dx | ALK Status | CD3 Status | CNS Disease | HCT-CI Score | Prior Auto HCT | First-line therapy (Disease Status)^a^ | Second-line therapy (Disease Status)^a^ | Third-line therapy (Disease Status)^a^ | Fourth-line therapy; additional therapies (Disease Status)^a^ | Disease Status Prior to AlloHCT | Donor source, cell type, HLA | HCT Conditioning | GVHD Prophylaxis | aGVHD  (NIH Grade) | cGVHD Dx (Peak Grade)  [Anatomic Location] | Best Disease Outcome Post-HCT | Post-HCT Progression/  Relapse | Outcome,  Last Follow-up Post-HCT |
| --- | --- | --- | --- | --- | --- | --- | --- | --- | --- | --- | --- | --- | --- | --- | --- | --- | --- | --- | --- | --- | --- |
| 1 | 1997 | 30 | III | Unk | Pos | No | 0 | No | CHOP + 4320 cGy (P) | DHAP (SD) | MINE (SD) |  | P | Sibling, PBSC, 10/10 | Bu, Cy | Csp, Mtx | Yes (2) | n/a | P | Yes | Relapse/Died,  0.4 yrs |
| 2 | 2000 | 25 | IV | Pos | Pos | Yes | 6 | No | EPOCH + Dac + 5080 cGy  (SD) | Dac (SD) |  |  | P | Unrelated, PBSC, 9/10 | Bu, Cy | Csp, Mtx | Yes (2) | n/a | P | Yes | Relapse/Died, 0.3 yrs |
| 3 | 2001 | 26 | II-A | Pos | Neg | No | 3 | No | CHOP (P) | ESHAP (P) | EPOCH + 3420 cGy (SD) |  | R | Sibling, PBSC, 10/10 | Cy, TBI 1200 cGy | Csp, Mtx | No | n/a | P | Yes | Relapse/Died, 0.2 yrs |
| 4 | 2003 | 44 | IV-A | Unk | Unk | No | 1 | Yes | m-BACOD (CR) | CHOP + AutoHCT (CR) | DHAP (P) | ICE + 4500 cGY (CR) | CR3 | Unrelated, PBSC, 10/10 | Cy, Flu, TBI 200 cGy | Csp, MMF | Yes (2) | Yes (unk)  [Skin, Oral, Eye, Skin, Lungs] | CR3 | No | NRM due to lung GVHD,  4 yrs |
| 5 | 2007 | 57 | II-B | Neg | Neg | No | 2 | No | Hyper-CVAD + 4500 cGy (CR) | ICE (CR) |  |  | CR2 | Sibling, PBSC, 10/10 | BEAM | Tac, Mtx | No | Yes (mild)  [Skin, Oral, Myofascial] | CR2 | No | CR/Died,  16.2 yrs, metastic sarcoma |
| 6 | 2008 | 32 | III-A | Pos | Neg | Yes; at relapse | 0 | Yes | Hyper-CVAD (PR) | CHOP (CR) | IT Ara-C (SD) | IT Ara-C + 3000 cGy (PR); ICE (PR); AutoHCT (SD) | PR | Unrelated, PBSC, 10/10 | Flu, TBI 200 cGy | Tac, MMF | No | n/a | P | Yes | Relapse/Died, 0.3 yrs |
| 7 | 2009 | 46 | II-A | Neg | Neg | No | 3 | No | CHOP + 3060 cGy (CR) | ICE (CR) |  |  | CR2 | Sibling, PBSC, 10/10 | Flu, TBI 200 cGy | Csp, MMF | Yes (2) | Yes  (moderate)  [Skin, Oral] | CR2 | No | CR Alive,  16.2 yrs |
| 8 | 2009 | 30 | II-BS | Pos | Neg | No | 4 | Yes | CHOP (CR) | ICE (PR) | GND + AutoHCT (CR) |  | CR2 | Unrelated, PBSC, 10/10 | Cy, Flu,  TBI 200 cGy | Csp, MMF | Yes (2) | Yes (severe)  [Skin, Oral] | CR2 | No | CR Alive,  15.6 yrs |
| 9 | 2009 | 44 | I-EB | Neg | Neg | No | 4 | Yes | CHOP + 4000 cGy (CR) | ICE (PR) | GND + AutoHCT (PR) |  | PR | Unrelated , PBSC, 10/10 | Flu, TBI 200 cGy | Csp, MMF | Yes (2) | Yes (moderate)  [Skin, Oral] | CR1 | No | CR Alive,  15.6 yrs |
| 10 | 2010 | 18 | III | Pos | Neg | No | 3 | Yes | Vin/Dox/6-MP (PR) | 6-MP (CR) | ICE + AutoHCT (CR) | BV (CR) | CR3 | Parent, BM, Haplo | Cy, Flu, TBI 200 cGy | PTCy, Tac, MMF | Yes (2) | Yes (severe)  [Gut, Oral] | CR3 | No | CR Alive,  15.3 yrs |
| 11 | 2011 | 62 | III | Neg | Pos | No | 2 | No | CHOP (CR) | BV (PR) | Belinostat (P) | Pralatrexate (PR) | PR | Child, PBSC, Haplo | Cy, Flu, TBI 200 cGy | PTCy, Csp, MMF | Yes (2) | Yes (severe) [Gut, Oral, Ocular] | CR2 | No | CR Alive,  14.6 yrs |
| 12 | 2011 | 45 | II-A | Neg | Neg | No | 3 | Yes | ABVD (P) | Hyper-CVAD + ICE (SD) | AutoHCT (PR) | 3600 cGY (PR) | PR | Unrelated , PBSC, 10/10 | Flu, TBI 200 cGy | Csp, MMF | Yes (2) | Yes (severe)  [Skin, Gut, Oral] |  | No | CR Alive,  14.6 yrs |
| 13 | 2012 | 15 | II-B | Neg | Neg | No | 0 | No | Vin, Dox (PR) | COPADM/CYM (P) | BV (P) | Vin + 3060 cGY (P); ICE (P); Pralatrexate (P); Temsirolimus (CR) | CR1 | Parent, BM, Haplo | Cy, Flu, TBI 200 cGy | PTCy, Tac, MMF | Yes (2) | n/a | CR1 | Yes | Relapse/Died,  0.5 yrs |
| 14 | 2012 | 33 | I | Pos | Neg | No | 3 | No | CHOP + 540 cGy (PR) | ICE (PR) | BV (P) | Oxaliplatin/ Cytarabine (P); Crizotinib (CR) | CR1 | Unrelated, Cord blood, 5/6 | Cy, Flu, TBI 1320 cGy | CSP, MMF | Yes (2) | No | CR1 | No | CR Alive,  13.4 yrs |
| 15 | 2012 | 12 | IV | Neg | Unk | No | 3 | No | COPAD (CR) |  |  |  | PR | Sibling, BM, 10/10 | BEAM | Tac, Mtx | Yes (2) | No | CR1 | No | CR Alive,  12.9 yrs |
| 16 | 2015 | 17 | III-B | Pos | Neg | No | 1 | No | Mitoxantrone/ Ara-C/Etop alternating with Mtx/Cy/ Dox (PR) | Crizotinib (CR) | BV (CR) |  | CR2 | Unrelated, PBSC, 10/10 | CY, TBI 1320 cGy | Tac, Mtx | Yes (2) | No | CR2 | No | CR Alive,  8.4 yrs |
| 17 | 2019 | 3 | IV | Pos | Pos | Yes; at relapse | 0 | No | COP (CR) | BV + Lorlatinib (CR) |  |  | CR2 | Unrelated, BM, 10/10 | Mel, Flu, TBI 400 cGy | Unk | Yes (2) | No | CR2 | No | CR Alive,  5.9 yrs |

Abbreviations: 6-MP, mercaptopurine; ABVD, doxorubicin, bleomycin, vincristine, dacarbazine; aGVHD, acute graft-vs-host disease; AlloHCT, allogeneic HCT; ALK, anaplastic lymphoma kinase; Ara-C, cytarabine; AutoHCT, autologous HCT; BEAM, carmustine, etoposide, cytarabine, melphalan; Bu, busulfan; BM, bone marrow; cGVHD, chronic graft-vs-host disease; BV, brentuximab vedotin; cGy, centigray; CHOP, cyclophosphamide; doxorubicin, vincristine, prednisolone; COP, cyclophosphamide, vincristine, prednisone; COPADM/CYM, cyclophosphamide, vincristine, prednisone, doxorubicin, methotrexate; CR, complete remission; Cy, cyclophosphamide; Csp, cyclosporine; DHAP, dexamethasone, cytarabine; Dac, daclizumab; Dox, doxorubicin; Dx, diagnosis; EPOCH, etoposide, prednisone, vincristine, cyclophosphamide, doxorubicin; ESHAP, etoposide, methylprednisolone, cytarabine, cisplatin; Etop, etoposide; Flu, fludarabine; GND, gemcitabine, navelbine, dexamethasone; GVHD, graft-vs-host disease; Haplo, haploidentical; HCT, hematopoietic cell transplant; HCT-CI, hematopoietic cell transplant comorbidity index; Hyper-CVAD, cyclophosphamide, vincristine, doxorubicin, dexamethasone; ICE, ifosfamide, carboplatin, etoposide; IT, intrathecal; m-BACOD, methotrexate, bleomycin, doxorubicin, cyclophosphamide, vincristine, dexamethasone Mel, melphalan; MINE, Mesna, ifosfamide, mitoxantrone, etoposide; MMF, mycophenolate mofetil; Mtx, methotrexate; m-BACOD, methotrexate, bleomycin, doxorubicin, cyclophosphamide, vincristine, dexamethasone; NRM, non-relapse mortality; P, progression; PBSC, peripheral blood stem cells; PR, partial response; PTCy, post-transplant cyclophosphamide; SD, stable disease; Siro, sirolimus; Tac, tacrolimus; TBI, total body irradiation; Unk, unknown; Vin, vincristine.

^a^Disease status at end of therapy denoted in parentheses.

**Supplementary Table 2 (S2).** Detailed Characteristics of Autologous Cohort

| ID | Year of HCT | Age at HCT  (Years) | Ann Arbor Stage at Dx | ALK Status | CD3 status | First-line therapy (Disease Status)^a^ | Second-line therapy (Disease Status)^a^ | Third-line therapy (Disease Status)^a^ | Fourth-line therapy; additional therapies (Disease Status)^a^ | Disease Status Prior to HCT | HCT Prep | Best Disease Outcome Post-HCT | Post-HCT Progression/  Relapse | Status, Follow-up post-HCT |
| --- | --- | --- | --- | --- | --- | --- | --- | --- | --- | --- | --- | --- | --- | --- |
| 1 | 1997 | 41 | II | Pos | Neg | Cy/Mtx/Vin (PR) | CHOP (CR) | EPOCH (CR) |  | CR2 | Bu, Mel, TT | CR | Yes | Relapse/Died, 6.4 yrs |
| 2 | 1997 | 43 | Unk | Unk | Unk | m-BACOD (PR) | CHOP (P) | DHAP (PR) |  | PR | Cy, Etop, TBI 1200 | P | Yes | P/Died, 0.4 yrs |
| 3 | 1999 | 23 | Unk | Neg | Neg | CHOP (CR) | DHAP (PR) |  |  | PR | Cy, Etop, TBI 1200 | CR2 | Yes | Relapse//Died, 0.5 yrs |
| 4 | 1999 | 54 | IV-A | Neg | Pos | ProMACE (PR) | Cy, Paclitaxel (CR) |  |  | CR1 | Cy, Etop, TBI 1200 | CR1 | No | CR/Died, 22.5 yrs unknown cause |
| 5 | 2001 | 49 | II-A | Neg | Pos | CHOP (CR) | DHAP (PR) | MIME (PR) |  | PR | Cy, Etop, TBI 1200 | CR2 | No | CR/Alive, 24.3yrs |
| 6 | 2003 | 64 | IV | Neg | Pos | CHOP (PR) | ICE (PR) |  |  | PR | Cy, Etop, TBI 1200 | CR1 | Yes | Relapse/Died, 1.2 yrs |
| 7 | 2003 | 58 | III | Neg | Neg | CEPP (PR) | DHAP (PR) | ICE (P) |  | P | Mel, Etop, Ara-C, Car | CR1 | Yes | Relapse/Died, 1.7 yrs |
| 8 | 2005 | 59 | III-B | Pos | Pos | CHOP (PR) |  |  |  | PR | Cy, Etop, TBI 1200 | CR1 | No | CR/Died, 18.8 yrs, respiratory failure/ metastatic prostate ca |
| 9 | 2006 | 45 | I | Neg | Neg | CHOP + 3600 cGy (PR) | ICE (PR) |  |  | PR | Bu, Mel, TT | CR1 | No | CR/Alive, 18.1yrs |
| 10 | 2009 | 51 | III-B | Neg | Neg | GVD (PR) + 8520 cGy | ICE (P) |  |  | P/Ref | Cy, Etop, TBI 1200 | P | Yes | Relapse/Died, 0.3 yrs |
| 11 | 2009 | 22 | II | Pos | Neg | CHOP (CR) | ICE (CR) |  |  | CR2 | Etop, Cy, TBI 1200 | CR | Yes | CR/Alive, 15.6 yrs |
| 12 | 2010 | 59 | IV-B | Neg | Pos | 3600 cGy+ CHOP + IT Mtx (PR) | ICE (CR) |  |  | CR1 | Cy, Etop, LI (400), TBI 1200 | CR | No | CR/Alive, 15 yrs |
| 13 | 2010 | 42 | I-B | Neg | Neg | CHOEP + 3240 cGy (CR) | BV (CR) |  |  | CR2 | Cy, Etop, TBI 1200 | CR | No | CR/Alive, 9 yrs |
| 14 | 2010 | 29 | IV | Neg | Pos | CHOP (PR) | ICE (CR) |  |  | CR1 | Cy, TBI 1200 | CR | Yes | Relapse/Died, 0.6 yrs |
| 15 | 2010 | 60 | IV-A | Neg | Pos | CHOP + 1500 cGy/ MINE (CR) |  |  |  | CR1 | Mel, Etop, Ara-C, Car | CR | No | CR/Died, 9.7 yrs, stroke |
| 16 | 2011 | 52 | I-E | Neg | Pos | CHOP + 4000 cGy (CR) | BV (CR) |  |  | CR2 | Cy, Etop, TBI 1200 | CR | Yes | Relapse/Died, 10.4 yrs |
| 17 | 2013 | 52 | IV-A | Neg | Neg | EPOCH (PR) | ICE (CR) |  |  | CR1 | Mel, Etop, Ara-C, Car | CR | No | CR/Alive, 11.5 yrs |
| 18 | 2013 | 39 | III-B | Neg | Neg | ABVD (P) | BEACOPP (P) | ICE (P) | BV (P); CHOEP (P) | P/Ref | Cy, Etop, LI (400), TBI 1200 | P | Yes | Relapse/Died, 0.5 yrs |
| 19 | 2014 | 62 | I-A | Neg | Neg | CHOP +3960 cGy (CR) | BV (CR) | ICE (SD) |  | CR2 | Mel, Etop, Ara-C, Car | CR | No | CR/Alive, 11.6 yrs |
| 20 | 2014 | 67 | IV-B | Neg | Pos | EPOCH (P) |  |  |  | P/Ref | Mel, Etop, Ara-C, Car | P | Yes | P/Died, 0.5 yrs |
| 21 | 2016 | 23 | II-B | Pos | Neg | EPOCH (CR) | BV/ Crizotinib (CR) |  |  | CR2 | Mel, Etop, Ara-C, Car | CR | Yes | Relapse/Died, 1.4 yrs |
| 22 | 2016 | 58 | IV-B | Neg | Neg | CHOP (CR) | BV (PR) | Pralatrexate (PR) | BV (CR) | CR2 | Mel, Etop, Ara-C, Car | CR | No | CR/Died, 7.3 yrs, PTCL-NOS/HLH |
| 23 | 2017 | 35 | IV-B | Neg | Pos | CHOP (CR) | BV (PR) | EPOCH (CR) |  | CR2 | Mel, Etop, Ara-C, Car | CR | No | CR/Alive, 1.4yrs |
| 24 | 2018 | 61 | IV-B | Pos | Pos | CHOP (CR) | BV/ICE | Gem/Car/Dex (P) | BV/Crizotinib; Pralatrexate; BV/Crizotinib (PR) | PR | Mel, Etop, Ara-C, Car | P | Yes | P/Died, 0.1 yrs |

Abbreviations: ABVD: doxorubicin, bleomycin, vincristine, dacarbazine; ALK: anaplastic lymphoma kinase; ara-C: cytarabine; BEACOPP: bleomycin, etoposide, doxorubicin, cyclophosphamide, vincristine, procarbazine, prednisone; bu: busulfan; BV: brentuximab vedotin; car: carmustine; CEPP: cyclophosphamide, etoposide, procarbazine, prednisone; cGy: centigray; CHOEP: cyclophosphamide; doxorubicin, vincristine, etoposide, prednisone; CHOP: cyclophosphamide; doxorubicin, vincristine, prednisone; CR: complete remission; Cy: cyclophosphamide; DHAP: dexamethasone, cytarabine; EPOCH: etoposide, prednisone, vincristine, cyclophosphamide, doxorubicin; etop: etoposide; GND: gemcitabine, navelbine, dexamethasone; GVHD: graft-vs-host disease; HCT: hematopoietic cell transplantation; HLH: hemophagocytic lymphohistiocytosis; ICE: ifosfamide, carboplatin, etoposide; IT: intrathecal; LI: localized irradiation; mel, melphalan; MIME: methyl-GAG, ifosfamide, methotrexate, etoposide; MINE: mesna, ifosfamide, mitoxantrone, etoposide; mtx: methotrexate; m-BACOD: methotrexate, bleomycin, doxorubicin, cyclophosphamide, vincristine, dexamethasone; P/Ref: progression/refractory; PR: partial response; ProMACE: prednisone, methotrexate, doxorubicin, cyclophosphamide, etoposide; PTCL-NOD: peripheral T cell lymphoma, not otherwise specified; SD: stable disease; TBI: total body irradiation; TT: thiotepa; unk: unknown; vin: vincristine.

^a^Disease status at end of therapy denoted in parentheses.
