# Supplementary material for: Autologous and allogeneic hematopoietic cell transplantation in children and adults with high-risk anaplastic large cell lymphoma
Source: Front Oncol. 2026 Apr 22;16:1785566. doi: 10.3389/fonc.2026.1785566 (PMC13147282; doi:10.3389/fonc.2026.1785566)
Supplement: Supplementary file 2 [file Table2.docx]

| **Supplementary Table 3 (S3).** Literature review of ALCL trials using autologous and/or allogeneic HCT | n | Age Range (years) | Years Transplanted | Conditioning | Disease Status at Transplant | OS | PFS/EFS/DFS | Comments |
| --- | --- | --- | --- | --- | --- | --- | --- | --- |
| **Autologous HCT,** Multicenter | | | | | | | | |
| Fanin et al. ^1^ | 64 ALCL | 3-53 | 1983-1996 | 17.2% TBI  81.2% Chemotherapy NS | 47% CR  28% PR  12.5% Ref | 70.3%  (5y) | PFS 56.4%  (5y) | EBMT centers.  75% responsive to initial therapy |
| Rodriguez  et al.^2^ | 115 PTL  (25 ALCL) | 13-72 | 1990-1999 | 43% BEAM  32% BEAC  12% TBI  9% CVB  4% other | 57% CR  38% PR  5% Ref | 56% (5y) | DFS 60% (5y) | GEL-TAMO registry. R/R patient cohort. no difference in CR, DFS, OS for ALCL histology vs other PTL |
| Deconinck et al.^3^ | 202 NHL  15 ALCL | 15-60 | 1990-1994 | BEAM | 73% CR  27% SD | 87% (5y) | EFS 87% (5y) | Prospective Groupe Ouest-est  d'eÂtude des LeuceÂmies et Autres Maladies du Sang (GOELAMS) study |
| Jantunen et al.^4^ | 37 PTL  (14 ALCL) | 16-68 | 1990-2001 | 59% BEAC  41% BEAM | 49% CR1/PR1  38% CR2/PR2 | 54% (all, 5y)  ALCL 85% (5y) | PFS 44% (all, 5y)  ALCL PFS 65% (5y) | Retrospective Finnish transplant centers’ study |
| Numata et al.^5^ | 39 PTL  (9 ALCL) | 16-68 | 1990-2005 | 82% MCEC  18% TBI | 59% CR1/PR1  18% Ref | 62.2% (5y) | PFS 60.6% (5y) | Retrospective FBMTG registry study |
| Domingo-Domènech et al.^6^ | 65 ALCL | 20-71 | 2010-2014 | 92% BEAM/BEAM-like | 78% CR/PR | 73% (3y) | PFS 64% (3y) | Retrospective EBMT center study. R/R patient cohort. 15% of patients received BV prior to autoHCT |
| Brink et al.^7^ | 28 | 18-65 | 1989-2018 | Various/Not reported | 22 CR | 96% (5y) | Not reported |  |
| **Autologous HCT,** Single Center | | | | | | | | |
| Song et al. ^8^ | 36 PTL  (9 ALCL) | 19-62 | 1987-2001 | Etop/Mel or Etop/Mel/TBI | 42% CR  50% PR | 48% (3y)  ALCL  78% (3y) | EFS 37% (3y)  ALCL EFS 67% (3y) | Retrospective R/R patient cohort from University of Toronto Autologous Blood and Marrow Transplant Program. |
| Chen et al.^9^ | 53 PTL  (18 ALCL) | 18-73 | 1988-2006 | 17% TBI  83% Chemotherapy NS | 28% CR1/PR1  49% CR2/PR2+  19% Ref | 48% (5y) | PFS 25% (5y) | Retrospective Stanford University Blood and Marrow Transplantation study. Unpublished data compared ALCL histology with other PTL, no survival difference. |
| **Allogeneic HCT**, Multicenter | | | | | | | | |
| Le Gouill et al. ^10^ | 77 PTL  (27 ALCL) | 12-61 | 1988-2006 | 66% TBI  26% RIC | 40% CR  30% PR  30% SD/PD/Ref | 57% (5y)  ALCL 55% (5y) | EFS 53% (5y)  ALCL EFS 48% (5y) | Retrospective SFGM-TC registry study. All patients received at least one line of therapy before alloHCT. Prior therapies included autoHCT in 25% cases. |
| Fukano et al. ^11^ | 38 ALCL | 9-30 | 1990-2010 | 21% RIC  79% MAC | 45% CR | RIC 100% (5y)  MAC 48.5% (5y) | RIC EFS 87.5% (5y)  MAC EFS 42.8% (5y) | Retrospective FBMTG registry study. R/R cohort. 26% with prior autoHCT. TRM RIC 0%, MAC 25.9% |
| Woessmann et al. ^12^ | 20 ALCL | 0-14 | 1991-2003 | 30% Etop/TT/ TBI  45% Etop/Csp/TBI | 60% CR  15% PR  15% PD  10% Not evaluable | Not stated | EFS 75% (3y) | Retrospective R/R pediatric cohort after first-line BFM type chemotherapy. 25% with prior autoHCT. |
| Dodero et al. ^13^ | 52 PTL  (11 ALCL) | 15-64 | 1999-2009 | 100% TT-based RIC | 75% PR/CR  25% Ref | 50% (5y)  ALCL 54% (5y) | PFS 40% (5y)  ALCL PFS 45% (5y) | Retrospective Italian centers study. 52% of patients received prior up-front autoHCT. |
| Strullu et al. ^14^ | 34 Alk+ ALCL | <18 | 1993-2011 | 91% MAC | 82% CR  18% detectable ALCL | 70% (5y) | EFS 58% | Retrospective French SFGM-TC study, CIR 18% (5y); TRM 24% (5y) |
| Domingo- Domènech et al. ^15^ | 44 ALCL | 19-67 | 2010-2014 | 48% RIC  52% MAC  36% TBI | 52% CR  25% PR  20% PD | 74% (3y) | PFS 65% (3y) | Retrospective EBMT study. Greater than half of patients received 3+ treatment regimens and 50% failed a prior autoHCT. 52% patients received BV prior to alloHCT. |
| Furqan et al. ^16^ | 182 ALCL | 18-76 | 2008-2019 | 64% RIC/NMA  36% MAC | 62% CR  30% PR  8% Resistant  1% unknown | 56% (5y) | PFS 41% (5y) | Retrospective CIBMTR study R/R patient cohort. 36% prior autoHCT. |
| Savage et al. ^17^ | 40 CD30+ Alk- ALCL (27 allo-HCT after A-CHOP; 13 alloHCT after CHOP) | 18-71 | 2013-2016 | NS | All with CR prior to HCT | - | A-CHOP tx PFS 76% (5y)  CHOP tx PFS 59% (5y) | Prospective ECHELON-2 trial exploratory analysis; Achieved CR prior to HCT; Cohorts too small for reliable comparative studies |
| **Mixed AutoHCT and AlloHCT** | | | | | | | | |
| Guilino-Roth, et al. ^18^ | 13 ALCL (8 Auto-HCT; 5 Allo-HCT) | <21 | 1982-2004 | NS | 8 in CR, 5 in PR | 83% (5y) | DFS 77% (5y) | Retrospective MSKCC study |
| Jagasia et al. ^19^ | 9 PTCL, 3 NK-T cell, 16 ALCL  (Auto-HCT 11; Allo-HCT; 5) | 8-54 | 1988-2002 | 64% Cy/Etop/TBI  36% Cy/BCNU/Etop/Cispl | 36.3% CR  54.5% PR | ALCL 80% (3y) | EFS 69% (3yr)  ALCL EFS 53% (3y) | Retrospective Single Center. Vanderbilt University Medical Center. Excluded primary cutaneous ALCL. |
| Gross et al. ^20^ | 182 R/R NHL  36 ALCL (Auto-HCT=24; Allo-HCT=12) | ≤18 | 1990-2005 | Auto-HCT: 39% TBI; 34% Bu/Cy or Cy/Etop; 27% other; Allo-HCT: 86% TBI, 2% Bu/Cy, 12% NS | ≥CR2 48% Auto-HCT, 51% Allo-HCT  R/PD Auto-HCT 52%, Allo-HCT 49% | - | ALCL Auto-HCT EFS 35% (5y)  ALCL Allo-HCT EFS 46% (5y) | Retrospective multicenter CIBMTR registry. R/R Pediatric patient cohort. Excluded prior autoHCT. |
| Knörr et al. ^21^ | 22 Auto-HCT  36 Allo-HCT | <22 | 2004-2014 | TBI for Allo-HCT or Busuflan based if <24mo; BEAM for Auto-HCT | Subdivided into risk groups | Auto-HCT 82% (5y)  Allo-HCT 83% | HCT after first relapse; Auto-HCT 44% (5y); Allo-HCT 82% (5y) | Prospective ALCL-Relapse study by European Inter-Group for Childhood Non-Hodgkin Lymphoma; Authors concluded early relapse does not benefit from Auto-HCT. |
|  |  |  |  |  |  |  |  |  |
| **Tandem AutoHCT and AlloHCT, Multicenter** | | | | | | | | |
| Satwani et al.^22^ | 3 ALCL | 2-13 | 2001-2011 | Auto: BCNU, Etop, Cy  Allo: Bu/Flu | 1 in CR, 2 in PR | 33% (2yr) | 1 relapsed and died of disease | Prospective multisite study evaluating tandem AutoHCT followed by AlloHCT for lymphoma |
| Marks et al.^23^ | 58 (10 Auto, 47 Allo, 1 Auto/Allo) | ≤21 | 2011-2022 | Multiple listed in manuscript supplemental file | 3 not in CR prior to Allo: 1 with unknown disease status at HCT, 1 in PR with post-HCT maintenance therapy, 1 in PR without post-HCT therapy | Of those in CR: Allo at 5yr 94%; Auto at 5yr 91% | Of those in CR: Allo 5yr EFS 64%; Auto 5yr EFS 55% | 56 received HCT after first relapse, 3 after ≥2 relapse; 8 received maintenance therapy post-HCT: 6 ALK inhibitor median 7.5 months (range 1.4-22.5) and 2 received BV for 2.1 and 6.3 months; at data cutoff 2 patients on maintenance therapy, no relapses in those received maintenance therapy post-HCT; 12 post-HCT relapses (4 Auto, 8 Allo), 4 died of TRM, 1 died unknown causes |
| Pereira et al.^24^ | 29 Allo, 19 Auto | <18 | 1999-2017 | Not listed | Not listed | Not listed | 5yr PFS for Allo 66% and 42% for Auto | 3 died from TRM related to HCT, 7 post-HCT relapses (2 received vinblastine, 1 received donor lymphocyte infusion and one died of progressive disease) |

*Abbreviations:*

allo: allogeneic; ALCL: anaplastic large cell lymphoma; auto: autologous; BEAC: carmustine, etoposide, cytarabine, and cyclophosphamide; BEAM: carmustine, etoposide, cytarabine, melphalan; BFM: Berlin-Frankfurt-Münster; bu: busulfan; MBV: brentuximab vedotin; CIBMTR: Center for International Blood and Marrow Transplant Research; CR: complete remission; Cy: cyclophosphamide; Cispl: cisplatin; DFS: disease free survival; EBMT: European Society for Bone Marrow Transplantation patient registry; EFS: event free survival; Etop: etoposide; FBMTG: Fukuoka Blood & Marrow Transplant Group; flu: fludarabine; GEL-TAMO: Grupo Español de Linfomas/ Trasplante Autólogo de Médula Ósea; HCT: hematopoietic cell transplantation, MAC: myeloablative conditioning; MCEC: ranimustine, carboplatin, etoposide, cyclophosphamide; mel: melphalan; NHL: non-Hodgkin Lymphoma; NS: not specified; OS: overall survival; PFS: progression free survival; PR: partial remission; PTL: peripheral T-cell lymphoma; R/R: recurrent and refractory; Ref: refractory disease; RIC: reduced intensity conditioning; SFGM-TC: Société Francophone de Greffe de Moelle et Thérapie Cellulaire; TBI: total body irradiation; TRM: transplanted related mortality; TT: thiotepa.

1. Fanin, R., Ruiz de Elvira, M. C., Sperotto, A., Baccarani, M. & Goldstone, A. Autologous stem cell transplantation for T and null cell CD30-positive anaplastic large cell lymphoma: analysis of 64 adult and paediatric cases reported to the European Group for Blood and Marrow Transplantation (EBMT). *Bone Marrow Transplant.* **23**, 437–442 (1999).
2. Rodríguez, J. *et al.* High-dose chemotherapy and autologous stem cell transplantation in peripheral T-cell lymphoma: the GEL-TAMO experience. *Ann. Oncol.* **14**, 1768–1775 (2003).
3. Deconinck, E. *et al.* Autologous stem cell transplantation for anaplastic large-cell lymphomas: results of a prospective trial. *Br. J. Haematol.* **109**, 736–742 (2000).
4. Jantunen, E. *et al.* Autologous stem cell transplantation in adult patients with peripheral T-cell lymphoma: a nation-wide survey. *Bone Marrow Transplant.* **33**, 405–410 (2004).
5. Numata, A. *et al.* Long-term outcomes of autologous PBSCT for peripheral T-cell lymphoma: retrospective analysis of the experience of the Fukuoka BMT group. *Bone Marrow Transplant.* **45**, 311–316 (2010).
6. Domingo-Domènech, E. *et al.* Autologous hematopoietic stem cell transplantation for relapsed/refractory systemic anaplastic large cell lymphoma. A retrospective analysis of the lymphoma working party (LWP) of the EBMT. *Bone Marrow Transplant.* **55**, 796–803 (2020).
7. Brink M,  Meeuwes FO, van der Poel MWM, Kersten MJ,  Wondergem M, Mutsaers PGNJ,  Böhmer LH,  Woei-A-Jin FJSH, Visser O, Oostvogels R, Jansen PM, Plattel W, Huls GA, Vermaat JSP, Nijland M; Impact of etoposide and ASCT on survival among patients aged <65 years with stage II to IV PTCL: a population-based cohort study. Blood 2022; 140 (9): 1009–1019.
8. Song, K. W., Mollee, P., Keating, A. & Crump, M. Autologous stem cell transplant for relapsed and refractory peripheral T-cell lymphoma: variable outcome according to pathological subtype. *Br. J. Haematol.* **120**, 978–985 (2003).
9. Chen, A. I., McMillan, A., Negrin, R. S., Horning, S. J. & Laport, G. G. Long-term results of autologous hematopoietic cell transplantation for peripheral T cell lymphoma: the Stanford experience. *Biol. Blood Marrow Transplant. J. Am. Soc. Blood Marrow Transplant.* **14**, 741–747 (2008).
10. Le Gouill, S. *et al.* Graft-versus-lymphoma effect for aggressive T-cell lymphomas in adults: a study by the Société Francaise de Greffe de Moëlle et de Thérapie Cellulaire. *J. Clin. Oncol. Off. J. Am. Soc. Clin. Oncol.* **26**, 2264–2271 (2008).
11. Fukano, R. *et al.* Successful outcome with reduced-intensity condition regimen followed by allogeneic hematopoietic stem cell transplantation for relapsed or refractory anaplastic large-cell lymphoma. *Int. J. Hematol.* **110**, 723–728 (2019).
12. Woessmann, W. *et al.* Allogeneic haematopoietic stem cell transplantation in relapsed or refractory anaplastic large cell lymphoma of children and adolescents – a Berlin–Frankfurt–Münster group report. *Br. J. Haematol.* **133**, 176–182 (2006).
13. Dodero, A. *et al.* Allogeneic transplantation following a reduced-intensity conditioning regimen in relapsed/refractory peripheral T-cell lymphomas: long-term remissions and response to donor lymphocyte infusions support the role of a graft-versus-lymphoma effect. *Leukemia* **26**, 520–526 (2012).
14. Strullu, M. *et al.* Hematopoietic stem cell transplantation in relapsed ALK+ anaplastic large cell lymphoma in children and adolescents: a study on behalf of the SFCE and SFGM-TC. *Bone Marrow Transplant.* **50**, 795–801 (2015).
15. Domingo-Domènech, E. *et al.* Allogeneic hematopoietic stem cell transplantation for patients with relapsed/refractory systemic anaplastic large cell lymphoma. A retrospective analysis of the Lymphoma Working Party of the European Society for Blood and Marrow Transplantation. *Bone Marrow Transplant.* **55**, 633–640 (2020).
16. Furqan, F. *et al.* Allogeneic haematopoietic cell transplant in patients with relapsed/refractory anaplastic large cell lymphoma. *Br. J. Haematol.* **200**, 54–63 (2023).
17. Savage, K. J. *et al.* Role of stem cell transplant in CD30+ PTCL following frontline brentuximab vedotin plus CHP or CHOP in ECHELON-2. *Blood Adv.* **6**, 5550–5555 (2022).
18. Giulino-Roth, L. *et al.* Ten-year follow-up of pediatric patients with non-Hodgkin lymphoma treated with allogeneic or autologous stem cell transplantation. *Pediatr. Blood Cancer* **60**, 2018–2024 (2013).
19. Jagasia, M. *et al.* Histology Impacts the Outcome of Peripheral T-Cell Lymphomas after High Dose Chemotherapy and Stem Cell Transplant. *Leuk. Lymphoma* **45**, 2261–2267 (2004).
20. Gross, T. G. *et al.* Hematopoietic stem cell transplantation for refractory or recurrent non-Hodgkin lymphoma in children and adolescents. *Biol. Blood Marrow Transplant. J. Am. Soc. Blood Marrow Transplant.* **16**, 223–230 (2010).
21. Knörr, F. *et al.* Stem Cell Transplantation and Vinblastine Monotherapy for Relapsed Pediatric Anaplastic Large Cell Lymphoma: Results of the International, Prospective ALCL-Relapse Trial. *J. Clin. Oncol. Off. J. Am. Soc. Clin. Oncol.* **38**, 3999–4009 (2020).
22. Satwani P, Jin Z, Martin PL, Bhatia M, Garvin JH, George D *et al.* Sequential myeloablative autologous stem cell transplantation and reduced intensity allogeneic hematopoietic cell transplantation is safe and feasible in children, adolescents and young adults with poor-risk refractory or recurrent Hodgkin and non-Hodgkin lymphoma. *Leukemia* 2015; **29**(2)**:** 448-455. e-pub ahead of print 20140618; doi: 10.1038/leu.2014.194
23. Marks LF,  Ritter V,  Agrusa J,  Kamdar KY,  Rivers J,  Gardner RA,  Ehrhardt MJ,  Devine KJ,  Phillips CA, Reilly AF,  August K,  Weinstein JL,  Satwani, Jon Forlenza CP,  Smith CM,  Greer C,  Afify Z, Carol H. Lin CH,  Belsky JA,  Ding H, Hoogstra D, Toner, K  Link MP,  Schultz, Lowe EJ,  Aftandilian C; Pediatric relapsed/refractory ALK+ anaplastic large cell lymphoma treatment and outcomes in the targeted drug era. Blood Adv2025; bloodadvances.2024014745.
24. Pereira, V., Barthoulot, M., Aladjidi, N., Contet, A., Dalle, J.-H., Dourthe, M. É., Garnier, N., Bruno, B., Leruste, A., Pellier, I., Simonin, M., Paillard, C., Verschuur, A., Ducassou, S., Lamant, L., Brugieres, L., Deley, M.-C. L., & Rigaud, C. (2025). Outcome of childhood ALK-positive anaplastic large cell lymphoma relapses: Real-life experience of the French Society of Pediatric Oncology (SFCE) cohort of 75 French children. *Pediatric Blood & Cancer*, *72*(1), e31397
